# Knowledge, Attitude, and Perceptions about In Vitro Fertilization (IVF) among Women of Childbearing Age in Cape Coast, Ghana

**DOI:** 10.1155/2022/5129199

**Published:** 2022-07-07

**Authors:** Samuel Kofi Arhin, Richard Tang, Aisha Hamid, Delali Dzandu, Bright Kwaku Akpetey

**Affiliations:** Department of Physician Assistant Studies, University of Cape Coast, Cape Coast, Ghana

## Abstract

**Background:**

Infertility impacts a lot of considerable negative social effects on the lives of infertile partners, especially females, who repeatedly experience the emotional sequelae of childlessness. The study's goal was to assess women of reproductive ages' awareness of IVF treatments, as well as their attitudes and misunderstandings about them in Cape Coast, Ghana.

**Methods:**

A total of 437 reproductive-age women in Cape Coast Metropolis were recruited using a simple random sampling approach for this Cross-Sectional Descriptive study. Data were collected with a semistructured interviewer-administered questionnaire and were analyzed using IBM SPSS version 26.0, and *p* ≤ 0.05 was considered significant.

**Results:**

The mean age was calculated to be 25.33 ± 0.066 years with a greater proportion, 65.7% within 15–24 years, 76.5% had no or had never had a child before. 93.4% were of the Christian faith, 66.8% were aware of IVF, and 74.8% think IVF offers hope. Although 41.4% believe it is not a natural procedure and 44.6% believe IVF children are normal but not natural. While 72.1% believe the treatment is very costly, and 40.7% believe it is not affordable or accessible. The majority believe IVF kids are legitimate (76.9%), and so should be welcomed by society (86.5%). The overall opinion of IVF service acceptability was 81.7% good. Seventy-two and three percent did not know whether IVF services are available in Cape Coast. Also, 48.1% were aware that IVF may result in pregnancy failure, with fewer than half (43.5%) believing it could be linked to genetic problems in the baby. The majority (60.4%) were willing to use IVF services, and 82.8% will utilize just their husband's sperm technique. While others may not want to undergo any form of IVF technique because they desire to conceive naturally (51.0%) and 22.4% may be unable to pay for it. Educational status and awareness of the availability of IVF services were factors that were significantly associated with their overall good perception of IVF services. Also, age, marital status, number of live children, occupation, educational status, awareness, and their overall perception were factors that are significantly associated with their preparedness to utilize IVF services.

**Conclusion:**

Overall, women's opinions of IVF and their readiness to use them were favorable, and they think it offers hope for their condition since they were well-informed about its forms and that infertility may be a result of several factors, all of which may need IVF services. It does not matter if it is difficult to obtain, expensive, or unavailable. It is recommended that the government collaborates with healthcare providers to investigate ways through the mass media in the drive to clear the misconceptions and improve the public understanding of the IVF procedure towards its utilization, thereby reducing the burden of childlessness and the resulting psychological disorders among couples, this has implications for joyful homes and societal growth.

## 1. Introduction

The failure of a spouse to conceive following a year of frequent unguarded sexual encounters is referred to as infertility [1–4] or owing to a person's inability to procreate, either individually or with his or her spouse [5]. The issues surrounding infertility and its alarming incidence are escalating worldwide [6]. Predominantly infertility has been documented in a variety of societies in Africa, and it is usually related to tubal blockage. Over 50% of the cases of infertility in low-income countries are reported in gynecological clinics [7].

Globally, about 10–15% of reproductive-age females are unable to naturally get pregnant within a year of unguarded sexual intercourse [8], and the phenomenon of infertility effect is between 60 and 168 million people worldwide [4], accounting for about 13 to 15% of couples [9]. Also, a study reported that infertility accounts for about one-third of the population in the sub-Saharan African region [10]. Female infertility cases account for about 55%, while the male factor accounts for around 30 to 40% of the total [11]. Due to the result of the rising global population, and the advanced age of marriage in Africa, the number of childless couples is also on the rise, though the prevalence of couple infertility is unique in diverse countries [12].

For the last few years, the use of ARTs services has grown manifolds and is now available as an alternative means for infertile couples in low- and high-income nations. Paradoxically, the decision for one to undertake an IVF procedure is only for privileged couples, indicating that many people will be unable to pay for IVF treatment. The financial barrier has prevented many people from utilizing IVF technology. It is, therefore, evidence that higher-income households will be able to pay for the services of IVF while families with lower incomes cannot afford them [13]. You again failed to justify this study; what is the knowledge gap?

### 1.1. Purpose of the Study

The study's goal was to assess women of childbearing age's awareness of IVF treatments, as well as their attitudes and misunderstandings about them in Cape Coast, Ghana.

## 2. Methods

### 2.1. Study Area and Population Selection

This descriptive cross-sectional study was carried out in Cape Coast metropolis, the administrative capital of the central region in Ghana. The study population was estimated from the total population of reproductive age females (15 to 49 years) of 49028 [14]. Using Yamane's Simplified Proportions Formula for calculating sample size and correcting for 10% nonresponse, a total of 437 reproductive-age females who stayed or had stayed for at least a year and with or without a child were recruited using a convenient, simple random sampling technique.

### 2.2. Data Collection

Data collection was done using a set of semistructured interviewer-administered questionnaires, adapted and modified from a previous study [7]. The study employed both primary quantitative and qualitative data collection procedures by the researchers through face-to-face semistructured interviewer-administered questionnaires and online Google forms. The research instrument consisted of six sections: Sociodemographic Characteristics of the Respondents, Respondents' source of information about IVF services, Respondents Perceptions and Misperceptions of IVF services, Respondents' Level of Awareness of IVF services, and Reasons for infertility that may need IVF services, forms of IVF and Respondents' Willingness to Use IVF Services. The Sociodemographic data of the female respondents consisted of Age, Marital status, Religion, Number of living children, Occupation, Tribe, and educational status.

### 2.3. Statistical Analysis

SPSS version 26.0 software for Windows was used to conduct the statistical analysis. For continuous numerical variables, descriptive analysis was done, with mean, frequency, and percentages, and standard deviation mean and standard error of the mean shown where applicable.

Also, inferential statistics were done to compare categorical variables, the Pearson Chi-square test was utilized, and multivariate analysis was performed using the nominal regression model to uncover predictive variables for perception and the desire to undergo IVF services among reproductive-age females in Cape Coast. The level of statistical significance (alpha) for the test was set at *p* ≤ 0.05.

### 2.4. Ethical Considerations

The University of Cape Coast's Institutional Review Board (IRB-UCC) granted Ethical clearance with approval ID (UCCIRB/CHAS/2021/150) to execute the study. Also, written informed consent was obtained, and made sure it was duly signed or accepted by each participant recruited for the study with the assurance that confidentiality of personal information will be guaranteed. Each respondent was given the right to partake in the study or to withdraw from the interview any time before completion if they thought their right of participation was violated upon further discussion. To ensure anonymity, the study's participants were also instructed not to reveal their identities throughout the interview or in filling out the online Google forms.

## 3. Results

### 3.1. Sociodemographic Characteristics of the Respondents

The social demographic characteristics of these reproductive-age women are described in Table 1. Among the 437 females, their mean age was calculated to be Mean ± SEM; 25.2 ± 0.066 years, with a greater proportion (*n* *=* 287 (65.7%) within the ages of 15–24 years. Over half of those who took part in the survey said they had no child, (*n* *=* 315 (76.5%), and less than one-fourth of the total respondents (*n* *=* 82 (18.8%) were married, while (*n* *=* 11 (2.5%) and (*n* *=* 10 (2.3%) were divorced and widowed, respectively. Almost all respondents were from the Christian faith (*n* *=* 408 (93.4%). A total of (*n* *=* 151(34.6%) respondents had secondary education, while (*n* *=* 200 (45.8%) had tertiary education, with (*n* *=* 40 (9.2%) having no formal education (Table 1).

### 3.2. Respondents' Source of Information about IVF Services

**Figure 1:**
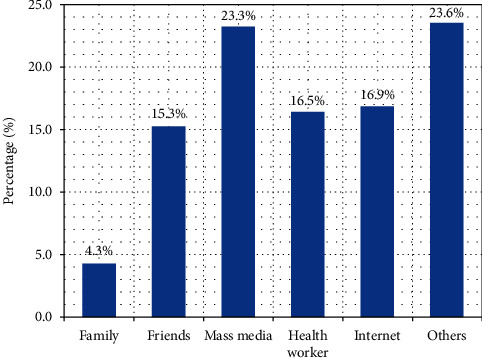
Respondents' source of information about IVF services.

More than half of females who responded (*n* = 292 (66.8%) said they had heard about IVF, with most (*n* *=* 103 (23.6%) obtaining their knowledge from the media and (*n* *=* 102 (23.3%) gaining their information from unspecified sources other than the mass media. Also, (*n* *=* 74 (16.9%) and (*n* *=* 72 (16.5%) of the respondent received their information from the Internet and from a health professional, respectively. In addition, (*n* *=* 67 (15.3%) heard from friends, while (*n* *=* 19 (4.3%) had theirs from a family member(s) (Figure 1).

### 3.3. Respondents' Perceptions and Misperceptions of IVF Services

IVF provides infertile couples hope, according to the majority of respondents (*n* *=* 327 (74.8%), although fewer than half (*n* *=* 181 (41.4%) believe it is not a natural procedure, and around (*n* *=* 196 (44.6%) believe IVF kids are normal but not natural (Table 2). The IVF treatment is too expensive (*n* *=* 315 (72.1%), not affordable, or not available (*n* *=* 178(40.7%), according to the majority of respondents, respectively. Most of the respondents think IVF babies are legitimate (*n* *=* 336 (76, 9%) and hence should be accepted (*n* *=* 378 (86.5%) by society. The overall perception of the acceptance of IVF service was positive (*n* *=* 357 (81.7%) among the majority of respondents (Table 2).

### 3.4. Respondents' Level of Awareness of IVF Services

The majority of respondents (*n* *=* 316 (72.3%) did not know whether IVF treatments services are accessible in the Cape Coast, and others (*n* *=* 210 (48.1%) were aware that IVF could lead to pregnancy failure, while less than half stated it could be associated Genetic abnormalities in the baby (*n* *=* 190 (43.5%) (Table 3).

### 3.5. The Reasons for Infertility That May Need IVF Services

Abnormal menses (*n* = 260 (59.5%), blocked tubes (*n* = 312 (71.4%), infection in the reproductive system of both women (*n* = 322 (73.7%) and men (*n* = 302 (69.1%), previous contraceptive method (*n* = 282 (64.5%), endocrine problems (*n* = 270 (61.8%), and marriage at an advanced age are all thought to be the cause of couple infertility by greater than half of the respondents (Table 4).

### 3.6. These Are Forms of IVF Services

Exactly half said the use of donor oocyte (*n* *=* 218 (49.9%) and donor sperm (*n* *=* 235 (53.8%) are types of IVF, whereas fewer than half said they use donor zygote (*n* *=* 184 (42.1%) and gametes preservation (*n* *=* 209 (47.8%) as forms of IVF (Table 5).

### 3.7. Respondents' Willingness to Use IVF Services

The majority of the respondents (*n* *=* 264 (60.4%) were ready to use IVF services. Greater than two-thirds of them will want to use only their husband's sperm method (*n* *=* 362 (82.8%) of IVF as much as they are willing to use. While a little above half may not want to use any IVF method: because they desire to conceive naturally (*n* *=* 223 (50.1%) and fewer than half (*n* *=* 98 (22.4%) will be unable to pay for it (Table 6).

### 3.8. Respondents' Background Characteristics by Their Perception of IVF Services

The information was analyzed using the Pearson Chi-Square test, and all statistical tests were run at a 5% level of significance. The educational status of the reproductive age females was statistically significant (*p*=0.032), related to their attitudes toward IVF services; those with tertiary (*n* *=* 172 (48.2%) and secondary (*n* *=* 112 (31.4%) education had a larger proportion of those with favorable perceptions. Similarly, the popular individuals who had heard about IVF (*n* *=* 259 (72.5%) had a good perception of the procedure (*p* ≤ 0.001) (Table 7).

### 3.9. Respondents' Background Characteristics by Their Preparedness to Use IVF

The Christian (*n* *=* 247 (93.6%) faith represented a majority of the respondents wanting to use IVF, although this was not statistically significant (*χ*^2^ (6) = 7.318, *p*=0.292). The age group of 15–24 years was represented by (*n* *=* 159 (60.2%) of the respondents who were eager to utilize IVF (*p* ≤ 0.001). Their preparedness to use the procedure was statistically significant among respondents with a single marital status (*n* *=* 190 (72.0%) and not having any living child (*n* *=* 179 (67.8%) (*p* ≤ 0.001 & *p* ≤ 0.001, respectively). There was a statistically significant link between knowledge (*n* *=* 206 (78.0%) and positive perception (*n* *=* 243 (92.0%), (*p* ≤ 0.001 & *p* ≤ 0.001, respectively) and the readiness to use IVF services if the need occurred. Also, a substantial number of students respondents (*n* *=* 156 (59.1%) by their occupation were ready to utilize IVF services if there is a necessity (*p* ≤ 0.001), and higher among those at the Tertiary level (*n* *=* 138 (52.3%) (Table 8).

### 3.10. Predictors of Respondent's Likelihood of Having a Positive Overall Perception of IVF Services

The findings of the relationship between the independent factors and participants' probability of having a positive overall perception of IVF services are presented in Table 9. Respondents between the ages of 15–24 years were 0.13 less likely to have a positive perception about IVF services in cape coast, though it was statistically significant (*p*=0.014), with a confident interval (lower limit 0.03 and upper limit 0.663). Also, those between the ages of 25–34 were 0.22 less likely to have a positive perception of IVF services, while those respondents between the ages of 35–44 years were 0.49 likely, neither to have a good nor bad perception of IVF services. Students and women in teaching were 1.52 and 1.50 respectively, more likely to have a positive perception of IVF services, but not statistically significant, while full-time housewives and businesswomen were 0.03 and 0.47 less likely to have a good perception of IVF respectively. Though full-time housewives' likelihood was statistically significant (*p*=0.009). Women respondents who had ever heard about the services of IVF in Cape Coast were 1.915 more likely to have a positive overall perception of IVF services, but this was not statistically significant (*p*=0.232), with a 95% confident interval greater than 1, i.e. (lower limit; 0.655 and upper limit; 5.559). The findings of the relationship between the independent factors and participants' probability of having a positive overall perception of IVF services are presented in (Table 9). Respondents between the ages of 15–24 years were less likely to have a positive perception of IVF services in cape coast, though it was statistically significant, (OR = 0.132, 95% CI (Lower limit 0.03 and upper limit 0.66), *p*=2.284), *p* ≤ 0.014). Also, those between the ages of 25–34 and 35–44 were less likely to have a positive perception of IVF services and not statistically associated; (OR = 0.220, 95% CI (Lower limit 0.05 and upper limit 0.1.06), *p*=0.059) and (OR = 0.497, 95% CI (Lower limit 0.094 and upper limit 2.627), *p*=0.410) respectively. Students and women in teaching were 0.520 and 0.500 more likely to have a positive perception of IVF services, but not statistically significant; (OR = 1.518, 95% CI (Lower limit 0.491 and upper limit 4.693), *p*=0.468) and (OR = 1.499, 95% CI (Lower limit 0.138 and upper limit 16.218), *p*=0.739) respectively, while full-time housewives and businesswomen were less likely to have a good perception of IVF services (OR = 0.033, 95% CI (Lower limit 0.003 and upper limit 0.434), *p* ≤ 0.01) and (OR = 0.473, 95% CI (Lower limit 0.144 and upper limit 1.558), *p*=0.219), respectively. Women respondents who had ever heard about the services of IVF in Cape Coast were 0.915 more likely to have a positive overall perception of IVF services, but this was not statistically significant (OR = 1.915, 95% CI (Lower limit 0.659 and upper limit 5.559).

### 3.11. Predictors of Respondents' Likelihood of Willingness to Utilize IVF Services

The findings of the association between the independent factors and respondents' possibility of having a desire to use IVF services are illustrated in Table 10. Female respondents without formal education were 2.012 likely to be willing to utilize the services of IVF if the need arises, though not statistically significant; those with primary education had a greater likelihood to use IVF services, and this was significant (OR = 11.41, *p*=0.019). At the same time, those with secondary educational level were less likely (OR = 0.442) to utilize IVF services and were also statistically significant (*p*=0.003). Women who had ever heard of IVF services had 2.629 increased odds of being eager to use it, but this was not statistically significant. Overall, the women respondent was 3.541 more probable to have a favorable opinion towards the utilization of the services IVF in cape coast, and this was statistically significant (*p* ≤ 0.001).

The text almost replicated the tables; it should rather be a summary of key findings in the table.

## 4. Discussion

Infertile couples now have a multitude of treatment choices because of ARTs [15]. According to the findings of this survey, a greater number of female respondents were mindful of IVF services (66.8%). This was comparable to findings in Sokoto, Nigeria, where over (70%) of the respondents knew of IVF services.

According to the findings, the majority of those who responded are familiar with the following possible causes of infertility: abnormal menses (59.5%), blocked fallopian tubes (71.4%), infection in the reproductive system of both women (73.7%) and men (69.1%), previous contraceptive use (64.5%), hormonal problems (61.8%), and marriage at an advanced age (57.4%). The high literacy rate of the respondents in the research area, which is known as the highly dominant educational institutions at all levels up to the university, might be a factor in the discrepancy. However, research conducted among university students in Ile-Ife, Nigeria, revealed that respondents' awareness of the various reasons for infertility is lacking, with more than half, 56%, having a poor understanding of infertility and just 44% having a better understanding [3, 16]. Also, a comparable study reported that due to the result of rising global population, and the advanced age of marriage in Africa, the number of childless couples is on the rise, though the prevalence of couple infertility is unique in diverse countries [4, 12]. Furthermore, another study reported contrasting findings, which stated that most African indigenes still think the causes of childlessness are rather spiritual and traditional than clinical, as seen in this current study [17].

The most popular source of information in this study was the mass media (23.6%), followed by unspecified sources other than the mass media (23.3%). The findings in Zaria, Awka, and Ibadan in Nigeria were comparable; however, the most popular information source in Tehran was from health centers. The disparity might be due to Nigeria's low IVF prevalence, where only a few clinics provide the services [18]. In addition, the source of information regarding ART's availability to clients had a significant effect on their choice to employ ART services. Electronic (Internet, TV, and radio) and print (magazines and newspapers) sources of information regarding the availability of ART services in Ghana are available, as are satisfied clients, friends, and members of social networks [8, 19, 20]. Also, in another study, it is reported that the helpful sources of information for infertility are written health center information on childlessness and the subsequent discussion with fertility clinic staff. Also, information sourced from family, colleagues, and support groups were comparable in this study [1, 21].

According to the findings, those without children made up the largest percentage of those surveyed (76.5%), and they think IVF offers hope (74.8%), and overall, 81.7% had a positive perception of IVF procedure, accounting for 66.8% of those who have heard of IVF services before the survey. These findings are comparable to a previous study in Saudi Arabia and Sokoto, Nigeria, where respondents' attitudes regarding ARTs were influenced by their awareness and the number of live children [7, 9]. Participants' general opinions regarding ART were increasingly encouraging as their degree of mindfulness of ART increased in research conducted at the University of Oxford, United States. This result might be due to the simple exposure effect, which states that simply being introduced to infertility management is enough to enhance reception and favorable views about them, unlike the opposite findings in cape coast metropolis, Ghana, where the public exposure to the IVF procedure is deficient [22].

The great majority of the respondents (72.3%) had no idea whether IVF treatments were accessible in the Cape Coast Metropolis, and another 48.1% were aware that IVF might result in pregnancy failure, with fewer than half (43.5%) believing it is connected to genetic problems in the baby. Comparably, the WHO reported that in certain places of the globe, particularly in poor and middle-income nations, these technologies are still unavailable, inaccessible, and costly to infertile women [4]. But an opposite finding was seen in a study conducted in Sokoto, Nigeria, which reported that the decreased number of women ready to employ IVF services was due to a lack of understanding and a poor view of the treatment [7]. This might be linked to a desire for a genetic association with their children, in addition to a desire to prevent marital strife over who is the biological parents of their expectant children [23].

Almost 50% of all women who took part in the survey may want to conceive naturally, while 22.4% may not want to use the procedure at all because they cannot afford it; this is similar to a study conducted in Nigeria and Pakistan, where 51.5% and 50%, respectively, of the respondents said they would employ the technique if they had to in the pursuit of their fertility wish [10, 24]. The higher rates in this current study could probably be owing to the educational status of the respondents and, likewise, the dominance of better educational establishments in the area. However, the result is greater than 59.3% found in Ibadan, Nigeria, and lower than 70.3% found in Saudi Arabia [7].

Regardless of most of the respondents being educated and well-informed, a substantial portion of the public still believes that IVF treatments are not available on Cape Coast. IVF is unknown to a majority of the women who answered the survey (72.3%). Despite the higher educational level, more than half were unsure whether IVF treatments were available. Also, 48.1% of the respondents believe infertility is connected to failure, while 43.5% believe it is connected to the baby's genetic problems. Though in other regions of Ghana, a growing number of reproductive clinics are springing up, which may suggest that despite the social, physical, and financial challenges connected with receiving ARTs treatments in the subregion, infertile women are yet seeking fertility wishes [4, 19].

Sixty and four percent showed they would be ready to use IVF procedures anytime. Whiles over two-thirds of them will prefer to utilize just their husband's sperm technique (82.8%). While slightly above half of those who took part in the survey may not want to use any IVF procedure because they prefer to conceive naturally (50.1%), and 22.4 percent believe they may be incapable of paying. In Cape Coast, the strong influence of unavailability of IVF services and the desire to use the husband's sperm may be the reasons given for the declining preparedness for women to utilize the IVF services, including the fact that most women listed the failure of the procedure and the associated genetic abnormalities as the grounds for which they may refuse IVF therapy. In contrast to this conclusion, Nigerian research found that those who follow certain religions or visit the house of worship are less inclined to embrace IVF. This suggests that some people would refuse IVF for the reason that they believed God would supply them with children without the need for any kind of ARTs [7, 25].

Exactly half stated they may use donor oocyte 49.9%, while 53.8% stated donor sperm, donor zygote 42.1%, or gametes preservation 47.8%, and the majority may want to use only their husband's sperm (82.8%) as a type of IVF procedure. However, depending on their reproductive status, they may accept any form if and only if it increases their likelihood of fertility desire being fulfilled. In line with this, a study from Nigeria found that up to half of their survey participants opted to use only their husband's sperm, and a similar one in Greece. Also, most authors from middle-income countries discovered that the majority of people would accept donor gametes for IVF therapy, provided it was kept hidden so that their offspring may be considered biological [10, 26]. All the development of ARTs had aided the conception in females of reproductive age to achieve their social ordeal of having children. Also, for the past twenty years, IVF services have been used safely and have proven to be beneficial among women who go in for any type of IVF procedure of their choice based on their state of infertility [15].

Educational status (*p*=0.032) and knowledge of the availability of IVF services (*p* ≤ 0.001) were factors that were significantly related to female respondents' overall good perception of IVF services, while Age (*p* ≤ 0.001), Marital status (*p* ≤ 0.001), Number of live children (*p* ≤ 0.001), Occupation (*p* ≤ 0.001), Educational status (*p* ≤ 0.001), Awareness (*p* ≤ 0.001), and Overall perception (*p* ≤ 0.001) were factors that were significantly associated with respondents' willingness to utilize IVF service [7, 27].

## 5. Conclusion

Overall, women's opinions of IVF services and their willingness to use them were favorable, and they think it offers hope for their condition since they were well-informed about its forms and that infertility may be a result of several factors, all of which may need IVF services. It does not matter if it is hard to get by, costly, or unavailable.

### 5.1. Study Limitations

One limitation of this present study is that the study area was dominated by major educational institutions, for which the majority of the study participants were between the ages of 15 and 24 years old and at various academic levels and may not have been married or had difficulty bearing children or in any way seeking fertility wish but may have given their opinions based on general perceptions and misconceptions. Rather than being in their 30 s and 40 s, married, or having some sort of fertility wish, and would like to share their own experiences with infertility and the use of IVF.

### 5.2. Recommendation

As a result, we, therefore, recommend that the government collaborates with healthcare providers to investigate ways through the mass media in the drive to clear the misconceptions and improve the public understanding of the in vitro fertilization procedure towards its utilization among individuals or couples with fertility wish in Cape Coast metropolis and Ghana as a whole. Thereby reducing the burden of childlessness and the resulting psychological disorders among couples. This has implications for joyful homes and societal growth.

### 5.3. Study Implications

The outcomes of this research have psychosocial and public health consequences. The findings imply that women of reproductive age are persistently pursuing their fertility wishes through various techniques, which have proven to be futile. Infertility has several significant negative social repercussions on infertile couples' lives, particularly females, who constantly endure the emotional consequences of childlessness due to the scarcity of fertility clinics with incorporated IVF services.

## Figures and Tables

**Table 1:** Respondents' sociodemographic characteristics.

Variable and responses	Frequency, *n* = 437	Percentage (%)
Age, years		
15–24	287	65.7
25–34	77	17.7
35–44	41	9.4
≥45	32	7.3
Mean age ± SEM; = 25.33 ± 0.066 years		
Marital status		
Single	334	76.4
Married	82	18.8
Divorced	11	2.5
Widow	10	2.3
Religion		
Islam	25	5.7
Christianity	408	93.4
Traditional	1	0.2
None	3	0.7
Number of living children		
0	315	76.5
1-2	52	11.9
3-4	43	9.8
≥5	27	6.2
Occupation		
Full-time housewife	4	0.9
Student	284	65.0
Businesswoman	92	21.1
Civil servant	5	1.1
Farming	5	1.1
Teaching	13	3.0
Others	43	7.8
Tribe		
Fante	227	51.9
Asante	74	16.9
Ewe	43	9.8
Dagaaba	7	1.6
Others	86	19.7
Educational status		
Informal	40	9.2
Primary	46	10.5
Secondary	151	34.6
Tertiary	200	45.8

**Table 2:** Respondents' perceptions and misperceptions of IVF services.

Variable and responses	Frequency, *n* = 437	Percentage (%)
Do you think IVF offers hope for infertile couples?		
Yes	327	74.8
No	20	4.6
Maybe	90	20.6
Do you think IVF is not a natural process?		
Yes	181	41.4
No	168	38.4
Maybe	88	20.1
What do you think about IVF babies?		
Normal and natural	195	44.6
Normal but not natural	196	44.9
Not normal and not natural	46	10.5
Do you think IVF is too expensive?		
Yes	315	72.1
No	23	5.3
Maybe	99	22.7
Do you think IVF is not affordable/accessible?		
Yes	178	40.7
No	133	30.4
Maybe	126	28.8
Do you think IVF babies should be accepted by society?		
Yes	378	86.5
No	21	4.8
Maybe	38	8.7
Do you think babies born through IVF are legitimate?		
Yes	336	76.9
No	39	8.9
Maybe	62	14.2
Overall perception about IVF		
Positive	357	81.7
Negative	80	18.3

**Table 3:** Respondents' level of qwareness of IVF services.

Variable and responses	Frequency, *n* = 437	Percentage (%)
Ever heard of IVF		
Yes	292	66.8
No	117	26.8
Maybe	28	6.4
Are IVF services available in cape coast metropolis?		
Yes	66	15.1
No	55	12.6
I do not know	316	72.3
IVF procedure may be associated with these problems		
Failure	210	48.1
Genetic abnormalities in the baby	190	43.5
Death	37	8.5
Respondents' source of information about IVF		
Family	19	4.3
Friends	67	15.3
Mass media	102	23.3
Health worker	72	16.5
Internet	74	16.9
Others	103	23.6

**Table 4:** These are some of the reasons for infertility that may need IVF.

Variable and responses	Frequency, *n* = 437	Percentage (%)
Abnormal menses		
Yes	260	59.5
No	77	17.6
I do not know	100	22.9
Blocked tubes		
Yes	312	71.4
No	38	8.7
I do not know	87	19.9
History of infections in the reproductive tract in women of reproductive age		
Yes	322	73.7
No	33	7.6
I do not know	82	18.8
History of infections in the reproductive tract in men		
Yes	302	69.1
No	38	8.7
I do not know	97	22.2
Previous use of contraceptive methods		
Yes	282	64.5
No	75	17.2
I do not know	80	18.3
Endocrine (hormones) problems		
Yes	270	61.8
No	51	11.7
I do not know	116	26.5
Marriage at an advanced age		
Yes	251	57.4
No	87	19.9
I do not know	99	22.7

**Table 5:** These are forms of IVF services.

Variable and responses	Frequency, *n* = 437	Percentage (%)
Use of donor oocyte (egg)		
Yes	218	49.9
No	117	26.8
I do not know	102	23.3
Use of donor sperm		
Yes	235	53.8
No	120	27.5
I do not know	82	18.8
Use of donor zygote		
Yes	184	42.1
No	143	32.7
I do not know	110	25.2
Preservation of gamete		
Yes	209	47.8
No	108	24.7
I do not know	120	27.5

**Table 6:** Respondents' preparedness to use IVF services.

Variable and responses	Frequency, *n* = 437	Percentage (%)
Willing to use IVF if the need arises		
Yes	264	60.4
No	75	17.2
I do not know	98	22.4
The method you will be willing to use		
Donor sperm	37	8.5
Only husband's sperm	362	82.8
Donor oocyte (egg)	13	3.0
Reasons for not wanting to use IVF		
Desire to conceive naturally	223	51.0
Religion	37	8.5
Culture	4	0.9
Will not be able to afford it	98	22.4
Others	75	17.2

**Table 7:** Chi-Square test of association between respondents' background characteristics by their perception of IVF services.

Variable and responses	Overall perception, *n* = 437		Test statistic & *p*-value
	Positive, *n* (%)	Negative, *n* (%)	
Age, years			
15–24	227 (63.6%)	60 (75.0%)	*df* = 3
25–34	66 (18.5%)	11 (13.8%)	*χ* ^2^ = 3.874
35–44	36 (10.1%)	5 (6.3%)	*V* = 0.094
≥45	28 (7.8%)	4 (5.0%)	*p*=0.275
Marital status			
Single	269 (75.4%)	65 (81.3%)	*df* = 3
Married	70 (19.6%)	12 (15.0%)	*χ* ^2^ = 1
Divorced	10 (2.8%)	1 (1.3%)	*V* = 0.062
Widow	8 (2.2%)	2 (2.5%)	*p*=0.641
Religion			
Islam	21 (5.9%)	4 (5.0%)	*df* = 3
Christianity	332 (93.0%)	76 (95.0%)	*χ* ^2^ = 1.013
Traditional	1 (0.3)	0 (0.0%)	*V* = 0.048
None	3 (0.8%)	0 (0.0%)	*p*=0.798
Number of living children			
0	256 (71.5%)	59 (73.8%)	*df* = 3
1-2	43 (12.0%)	9 (11.3%)	*χ* ^2^ = 0.894
3-4	37 (10.4%)	6 (7.5%)	*V* = 0.045
≥5	21 (5.9%)	6 (7.5%)	*p*=0.827
Occupation			
Full-time housewife	2 (0.6%)	2 (2.5%)	*df* = 6 *χ*^2^ = 5.188 *V* = 0.109 *p*=0.520
Student	230 (64.4%)	54 (67.5%)	
Businesswoman	75 (21.0%)	17 (21.3%)	
Civil servant	5 (1.4%)	0 (0.0%)	
Farming	4 (1.1%)	1 (1.3%)	
Teaching	12 (3.4%)	1 (1.3%)	
Others	29 (8.1%)	5 (6.3%)	
Educational status			
Informal	34 (9.5%)	6 (7.5%)	*df* = 3
Primary	39 (10.9%)	7 (8.8%)	*χ* ^2^ = 8.778
Secondary	112 (31.4%)	39 (48.8%)	*V* = 0.142
Tertiary	172 (48.2%)	28 (35.0%)	*p*=0.032^*∗*^
Ever heard of IVF			
Yes	259 (72.5%)	33 (41.3%)	*df* = 2 *χ*^2^ = 33.794 *V* = 0.278 *p* ≤ 0.001^*∗*^
No	75 (21.0%)	42 (52.5%)	
Maybe	23 (6.4%)	5 (6.3%)	

*df* = degree of freedom, *χ*2 = Pearson Chi-Square, *V* = Cramer's V, *p*-value, ^*∗*^ Significance at *p* ≤ 0.05.

**Table 8:** Chi-Square test for the relationship between respondents' background characteristics by their preparedness to utilize IVF services.

Variable & responses	Willingness to use IVF, *n* = 437			Test statistic & *p*-value
	Yes, *n* (%)	No, *n* (%)	I do not know, *n* (%)	
Age, years				
15–24	159 (60.2%)	46 (61.3%)	82 (83.7%)	*df* = 6
25–34	57 (21.6%)	9 (12.0%)	11 (11.2%)	*χ* ^2^ = 26.931
35–44	29 (11.0%)	9 (12.0%)	3 (3.1%)	*V* = 0.176
≥45	19 (7.2%)	11 (14.7%)	2 (2.0%)	*p* ≤ 0.001^*∗∗*^
Marital status				
Single	190 (72.0%)	51 (68.0%)	93 (94.9%)	*df* = 6
Married	62 (23.5%)	17 (22.7%)	3 (3.1%)	*χ* ^2^ = 28.395
Divorced	7 (2.7%)	4 (5.3%)	0 (0.0%)	*V* = 0.180
Widow	5 (1.9%)	3 (4.0%)	2 (2.0%)	*p* ≤ 0.001^*∗∗*^
Religion				
Islam	15 (5.7%)	67 (89.3%)	4 (4.1%)	*df* = 6
Christianity	247 (93.6%)	6 (8.0%)	94 (95.9%	*χ* ^2^ = 7.318
Traditional	0 (0.0%)	1 (1.3%)	0 (0.0%)	*V* = 1.092
None	2 (0.8%)	1 (1.3%)	0 (0.0%)	*p*=0.292
Number of living children				
0	179 (67.8%)	50 (66.7%)	86 (87.8%)	*df* = 6
1-2	41 (15.5%)	6 (8.0%)	5 (5.1%)	*χ* ^2^ = 21
3-4	28 (10.6%)	12 (16.0%)	3 (3.1%)	*V* = 0.157
≥5	16 (6.1%)	7 (9.3%)	4 (4.1%)	*p* ≤ 0.001^*∗∗*^
Occupation				
Full-time housewife	2 (0.8%)	2 (2.7%)	0 (0.0%)	*df* = 12 *χ*^2^ = 32.536 *V* = 0.193 *p* ≤ 0.001^*∗∗*^
Student	156 (59.1%)	43 (57.3%)	85 (86.7%)	
Businesswoman	65 (24.6%)	20 (26.7%)	7 (7.1%)	
Civil servant	4 (1.5%)	0 (0.0%)	1 (1.0%)	
Farming	4 (1.5%)	1 (1.3%)	0 (0.0%)	
Teaching	10 (3.8%)	1 (1.3%)	2 (2.0%)	
Others	23 (8.7%)	8 (10.7%)	3 (3.1%)	
Educational status				
Informal	22 (8.3%)	15 (20.0%)	3 (3.1%)	*df* = 6
Primary	39 (14.8%)	6 (8.0%)	1 (1.0%)	*χ* ^2^ = 55.868
Secondary	65 (24.6%)	31 (41.3%)	55 (56.1%)	*V* = 0.253
Tertiary	138 (52.3%)	23 (30.7%)	39 (39.8%)	*p* ≤ 0.001^*∗∗*^
Ever heard of IVF				
Yes	206 (78.0%)	42 (56.0%)	44 (44.9%)	*df* = 4 *χ*^2^ = 41.255 *V* = 0.217 *p* ≤ 0.001^*∗∗*^
No	47 (17.8%)	25 (33.3%)	45 (45.9%	
Maybe	11 (4.2%)	8 (10.7%)	9 (9.2)	
The overall perception of IVF				
Positive	243 (92.0%)	45 (60.0%)	69 (70.4%)	*df* = 2 *χ*^2^ = 50.863 *V* = 0.341 *p* ≤ 0.001^*∗∗*^
Negative	21 (8.0%)	30 (40.0%)	29 (29.6%)	

*df* = degree of freedom, *χ*2 = Pearson Chi-Square, *V* = Cramer's V, *p*-value, ^*∗*^ Significance at *p* ≤ 0.05.

**Table 9:** Respondents' likelihood of having a positive overall perception about IVF services.

Variable & response	Adjusted odds ratio (AOR)	95% CI for odds ratio (OR)		*p*-value
		Lower	Upper	
Age, years				
15–24	0.132	0.026	0.663	0.014^*∗*^
25–34	0.220	0.046	1.058	0.059
35–44	0.497	0.094	2.627	0.410
Occupation				
Student	1.518	0.491	4.693	0.468
Full-time housewife	0.033	0.003	0.434	*p* ≤ 0.01^*∗*^
Businesswoman	0.473	0.144	1.558	0.219
Farming	0.166	0.011	2.587	0.200
Teaching	1.499	0.138	16.218	0.739
Ever heard of IVF				
Yes	1.915	0.659	5.559	0.232

^*∗*^Significance at *p* ≤ 0.05.

**Table 10:** Likelihood of participants' preparedness to use IVF services.

Variable & responses	Adjusted odds ratio (AOR)	95% CI for odds ratio (OR)		*p*-value
		Lower	Upper	
Educational status				
Informal	2.012	0.559	7.236	0.284
Primary	11.471	1.492	88.194	0.019^*∗*^
Secondary	0.442	0.258	0.756	0.003^*∗*^
Have you ever heard of IVF?				
Yes	2.629	0.991	6.976	0.052
Overall perception				
Positive	3.541	1.787	6.980	*p* ≤ 0.001^*∗∗*^

^*∗*^ Significance at *p* ≤ 0.05

## Data Availability

The data used to support the findings of this study are included within the article.
